# Effect of the COVID-19 Mitigation Measure on Dental Care Needs in 17 Countries: A Regression Discontinuity Analysis

**DOI:** 10.3389/fpubh.2022.890469

**Published:** 2022-05-31

**Authors:** Xing Qu, Chenxi Yu, Qingyue He, Ziran Li, Shannon H. Houser, Wei Zhang, Ding Li

**Affiliations:** ^1^Institute of Hospital Management, West China Hospital, Sichuan University, Chengdu, China; ^2^College of Economics and Management, Sichuan Normal University, Chengdu, China; ^3^Southwest Medical University, Chengdu, China; ^4^Center of Health Care Management, Chengdu First People's Hospital, Chengdu Integrated TCM & Western Medicine, Chengdu, China; ^5^School of Public Finance and Taxation, Southwestern University of Finance and Economics, Chengdu, China; ^6^Department of Health Services Administration, University of Alabama at Birmingham, Birmingham, AL, United States; ^7^West China Biomedical Big Data Center, Med-X Center for Informatics, Sichuan University, Chengdu, China; ^8^Institute of Development Studies, Southwestern University of Finance and Economics, Chengdu, China

**Keywords:** COVID-19, oral health, Google Trends, regression discontinuity analysis, global health care, health service

## Abstract

**Objectives:**

The effect of COVID-19 mitigation measures on different oral health care needs is unclear. This study aimed to estimate the effect of COVID-19 mitigation measures on different types of oral health care utilization needs and explore the heterogeneity of such effects in different countries by using real-time Internet search data.

**Methods:**

Data were obtained from Google Trends and other public databases. The monthly relative search volume (RSV) of the search topics “toothache,” “gingivitis,” “dentures,” “orthodontics,” and “mouth ulcer” from January 2004 to June 2021 was collected for analysis. The RSV value of each topics before and after COVID-19 was the primary outcome, which was estimated by regression discontinuity analysis (RD). The effect bandwidth time after the COVID-19 outbreak was estimated by the data-driven optimal mean square error bandwidth method. Effect heterogeneity of COVID-19 on dental care was also evaluated in different dental care categories and in countries with different human development index (HDI) rankings, dentist densities, and population age structures.

**Results:**

A total of 17,850 monthly RSV from 17 countries were used for analysis. The RD results indicated that advanced dental care was significantly decreased (OR: 0.63, 95% CI: 0.47–0.85) after the COVID-19 outbreak, while emergency dental care toothache was significantly increased (OR: 1.54, 95% CI: 0.99–2.37) 4 months after the COVID-19 outbreak. Compared to the countries with low HDI and low dentist density, the effect was much more evident in countries with high HDI and high dentist density.

**Conclusions:**

COVID-19 mitigation measures have different effects on people with various dental care needs worldwide. Dental care services should be defined into essential care and advanced care according to specific socioeconomic status in different countries. Targeted health strategies should be conducted to satisfy different dental care needs in countries.

## Introduction

The coronavirus disease 2019 (COVID-19) pandemic has unpredictably and continuously disrupted the delivery of global health care utilization and disrupted essential health services in many countries (1). During the beginning of the pandemic, worldwide lockdown, quarantine, and redistribution of medical resources profoundly impacted the health care utilization of patients with chronic or urgent diseases. One England study reported that the substantial increases in the number of avoidable cancer deaths were expected due to diagnostic delays due to the COVID-19 pandemic (2). A US study showed that the COVID-19 pandemic had affected US psychiatry physicians by raising personal, financial, and ethical concerns (3). Daily increasing cases and deaths in the first wave of COVID-19 at the beginning of 2020 have led to fearing COVID-19 in many countries, and the number of patients who needed and sought emergency medical care services sharply dropped (4). Some patients with severe disease might even experience additional mortality if left untreated (5).

Oral disease is one of the most prevalent chronic diseases globally but is usually neglected (6). More than 3.5 billion people worldwide suffer from untreated dental caries or another oral condition (7). People with oral conditions would like to search online for similar symptoms or disease information on the Internet to relieve symptoms (8, 9). Therefore, this large volume of data generated from online searching behaviors could be analyzed following the concepts of infodemiology and infoveillance, first defined by Eysenbach (10), to identify previous knowledge and concerns of health seekers on a specific issue and to support health strategy planning (11). For example, interest in dental trauma, broken teeth, chipped teeth, knocked-out teeth, avulsed teeth and oral and maxillofacial surgery has shown a general increase in recent years (12, 13). In the dental health field, the most popular queries were markedly associated with symptoms and treatments, with little interest in prevention (14, 15). This phenomenon may be more pronounced during the pandemic. During the COVID-19 pandemic, it is a challenge for many people who seek regular dental examinations and dental cleaning appointments and those with more serious oral health problems to receive dental care in person, since many dental offices worldwide closed during the spring of 2020, given the risk of virus transmission (16). Interest in toothache-related digital information increased significantly after restriction measures were implemented in most countries (17). The search terms “bruxism,” “molars,” “toothache,” “dentist,” and “staying at home” were also increased (18, 19).

People with different oral conditions may have different online health information-seeking motivations and goals during the pandemic. To identify the individual goals of health information seeking, there are many theories to explain the context in which the search for information takes place. The broad and well-known concept of coping encompasses an individual's efforts to prevent or deal with distress, harm, or threat (20). Following the theory of problem- and emotion-focused coping initially proposed by Folkman and Lazarus (21), the goal of information-seeking between people with toothache and orthodontics-need was supposed to be different. People with toothache may tend to search for information about symptom relief or solution (22), while people with orthodontic needs may tend to compare dentists or treatment consequences. In addition, social determinants, including health culture, dentist supply (23), socioeconomic status (24), and aging structure (25), may also impact dental care service utilization, which may impact online health information-seeking behaviors. Analyzing this information may help us to understand the changes in dental health needs during the pandemic.

This study aimed to identify the effect of COVID-19 mitigation measures on different categories of dental care needs, evaluate the influence duration, and explore the heterogeneity of such effects in countries with different characteristics with Google Trends search topics by using a quasi-experimental method.

## Methods

### Data Source and Variable Measures

#### Relative Search Volume of Dental Care

This longitudinal retrospective study evaluated dental care-related computational metadata using Google Trends. After reviewing related articles and consulting senior dental experts, five search topics were included (26–29). The relative search volume (RSV) and the main related queries were obtained from the topics “Toothache—Search term,” “Gingivitis—Search term,” “Dentures—Search term,” “Orthodontics—Search term,” and “Mouth ulcer—Search term,” adopting the inclusion criteria of “all categories and sources,” between Jan 2010 and June 2021. The RSV indicates the proportion between the search volume of a specific query by the volume of overall queries performed by users on Google Search, normalized by the maximum value observed in a timeline (RSV = 0–100) and presented on a weekly or monthly basis. Only the countries with complete RSV information during each year were included in the analysis.

Dental care topics were divided into emergency dental care, basic dental care, and advanced dental care for a better understanding of the effect of COVID-19 on different dental care types. Although agreement on the concepts and definition of urgent and basic care is missing in dentistry, particularly in the COVID-19 pandemic context (30), following the concept of a previous study (31), we attempted to classify “toothache,” “gingivitis,” and “mouth ulcers” as basic dental care and “dentures” and “orthodontics” as advanced dental health care. Toothaches represent emergency dental care in basic dental care.

#### Human Development Index

The human development index (HDI) is a social indicator associated with dental services (32). The HDI is a summary measure of average achievement in key dimensions of human development: a long and healthy life, being knowledgeable and having a decent standard of living. The HDI is the geometric mean of normalized indices for each of the three dimensions, drawn from the United Nations Development Programme open database (33). Having a higher HDI means a higher standard of living. The HDI was ranked “very high,” “high,” “medium,” and “low” in each country by the UNDP. In this study, we set the original “very high” and “high” rank as the “High HDI group” and “medium” and “low” as the “Low HDI group” in the subgroup analysis.

#### Dentist Density

A lack of sufficient dental service providers is one of the barriers to accessing oral health care (23). Dentist density is defined as the number of dentists per 1,000 population in each country by the World Health Organization (WHO), which describes the convenience of dental care access. We used annual statistical data of dentist density drawn from the WHO from 2006 to 2020 to represent dental service providers. This study categorized the countries with a higher dentist density than the median of sum into the high dentist density group.

#### Population Age Structure

Dental care is different among populations with different age structures (25). The population age structure was drawn from World Bank demographic data from 2006 to 2019 (34). Following the definition of aged countries from the World Bank, we considered countries with a population over 65 years old above 14% as aged countries (35), while 65 years old below and equal to 14% were considered non-aged countries.

### Study Design

Sharp regression discontinuity (RD) was used to analyze the effect of the COVID-19 mitigation measures on dental care needs. RD is a quasi-experimental study design that identifies causal effects by deterministically exploiting a treatment assignment practice based on a continuously measured variable (36, 37). In this study, let *L* represent the treatment variable. *L* = 1 means the mitigation measures of COVID-19 (and *L* = 0). After March 2020, many countries imposed a national lockdown as a mitigation measure for COVID-19 to control coronavirus spread.

Let *C* represent the potential outcome (RSVs). For each country, we defined the potential outcome variables *C*^1^ and *C*^0^, corresponding to *L*^1^ and *L*^0^. The difference in dental disease RSVs before and after the COVID-19 outbreak was:


$$\begin{array}{l} (C/L=1)-E(C/L\;=\;0)\;=\frac{E(C^{1}-C^{0}/L\;=\;1)}{\tau }\\ \;+\;\frac{E(C^{1}/L\;=\;1)\;-\;E(C^{0}/L\;=\;0)}{\varepsilon } \end{array}$$

$\frac{E\left(C^{1}-C^{0}/L\;=\;1\right)}{\tau }$ is the average treatment-on-the-treated effect of RSVs in various countries. Such an effect depends on the impact of COVID-19 pandemic mitigation measures. $\frac{E\left(C^{1}/L\;=\;1\right)\;-\;E\left(C^{0}/L\;=\;0\right)}{\varepsilon }$ is selection bias, which summarizes all kinds of potential factors correlated with RSVs. Such bias should tend to zero when we restrict our sample to applicants close enough to the cutoff, while the incidence of dental healthcare still changes (discontinuously) at the cutoff. Therefore, comparing dental healthcare within a sufficiently narrow bandwidth of the cutoff but on opposite sides of it identifies the treatment effect of COVID-19 mitigation measures.

Let *X* represent the forcing variable, which denotes the timing of the pandemic outbreak with *X* = 0 at the cutoff, so *L* = 1 after *X* = 0 is a “treatment assignment” that is equal to 1 for RSVs after the cutoff. We set March 2020 as the cutoff point because from March 1st, 2020 to April 1st, 2020, new cases of COVID-19 sharply increased from 1,734 to 57,655. In March 2020, the World Health Organization declared COVID-19 to be a global pandemic (38). Then, the difference in limits $\underset{X\to 0}{\lim}\left[E\left(C/X>0\right)-E\left(C/X<0\right)\right]$ identifies the effect of *L* on RSVs near the cutoff. The bandwidth of all dental care services around March 2020 was determined using the data-driven optimal mean square error bandwidth method (39).

### Statistical Analysis

The descriptive analysis presented the characteristics of the participants against discount eligibility. Then, we adopted the RD approach to estimate the effect of the COVID-19 mitigation measures on different categories of dental care needs during a specific period. Graphical analysis was used to describe the RSV changes on both sides of the cutoff timing, conditional on the fixed effect of timing, country, and dental diseases. We also conducted placebo tests using outcomes that occurred during March 2019 and thus could not have been affected by the COVID-19 outbreak. A *t*-test was performed to demonstrate the differences in RSV between 12 months before and 12 months after COVID-19 onset through *P*-Values. Then, we estimated the heterogeneity of the COVID-19 impact on dental care needs among countries with different HDIs, dentist densities, and population age structures in the subgroup analysis. Odds ratios (ORs) and 95% confidence intervals (95% CIs) were reported. *P* < 0.05 was considered statistically significant. All analyses were performed by STATA 14.0 (Stata Corporation, College Station, TX, United States).

## Results

### Descriptive Information

**Figure 1:**
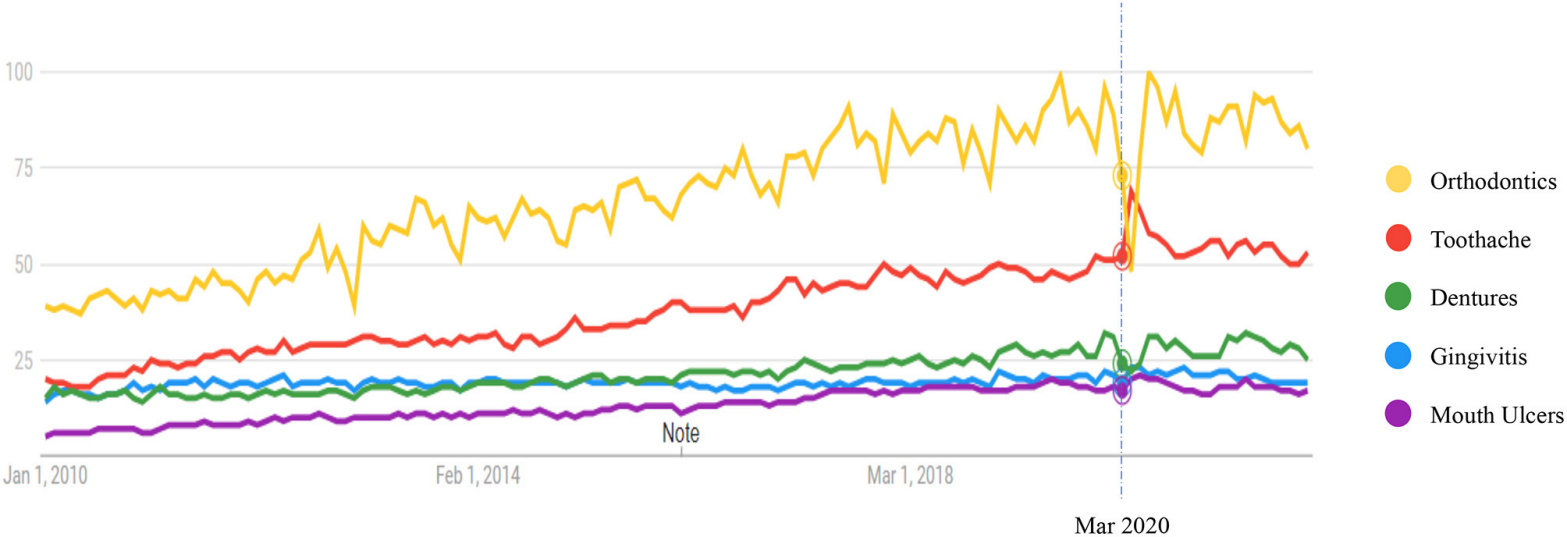
Search volume index of different dental care keywords using Google Trends, 2010–2021.

Figure 1 shows the RSV trends of five search topics in all countries from Jan 1st 2010 to Dec 31st 2021. Seventeen countries with sufficient monthly RSV information remained for analysis. These countries included Australia, Canada, France, Germany, India, Ireland, Malaysia, New Zealand, Pakistan, the Philippines, Saudi Arabia, Singapore, South Africa, Spain, the United Arab Emirates, the United Kingdom, and the United States of America. During 2010–2021, the RSV curves of “orthodontics,” “toothache,” and “dentures” gradually increased over time, while the curves of “gingivitis” and “mouth ulcers” seemed flat. However, during March 2020, the curve showed a fluctuation.

Table 1 reports descriptive information on the RSVs, HDI, dental density, and population age structure for the overall sample. A total of 17,850 RSV samples were analyzed in the total sample, and each dental type contained 3,570 samples. The mean RSVs of toothache, gingivitis, dentures, orthodontics, and mouth ulcers were 13.91, 9.13, 9.94, 16.14, and 7.74, respectively. The HDI mean was 0.767, and the mean dentist density was 4.093. The mean population aged >65 years was 10.47%.

**Table 1:** Descriptive information of search volume index, human develop index, dentist's density, and age structure.

	**(1)**	**(2)**	**(3)**	**(4)**	**(5)**
	***N***	**Mean**	**SD**	**Min**	**Max**
**Search volume index**
Total samples	17,850	11.38	14.21	0	100
Toothache	3,570	13.91	15.16	0	100
Gingivitis	3,570	9.13	11.43	0	100
Dentures	3,570	9.94	11.54	0	100
Orthodontics	3,570	16.14	19.39	0	100
Mouth ulcer	3,570	7.74	9.48	0	100
Human develop index	14,400	0.767	0.16	0.33	0.96
HDI rank	14,400	61.06	57.43	2.00	185
Dentist's density	9,120	4.09	2.46	0.02	8.58
Percent of aged >65	16,320	10.47	6.12	0.69	21.56

*HDI, human development index. SD, standard deviation*.

Table 2 shows the country category information in different groups of HDIs, dentist densities, and population age structure classifications. For instance, Australia is a country with high HDI, high dentist density and an aged population.

**Table 2:** Country categories in HDI, dentist density, and population age structure.

	**HDI**		**Dentist density**		**Population age structure**	
	**High**	**Low**	**High**	**Low**	**Aged**	**Non-aged**
Australia	√		√		√	
Canada	√		√		√	
France	√		√		√	
Germany	√		√		√	
India		√		√		√
Ireland	√		√		√	
Malaysia	√			√		√
New Zealand	√		√		√	
Pakistan		√		√		√
Philippines		√		√		√
Saudi Arabia	√			√		√
Singapore	√			√		√
South Africa		√		√		√
Spain	√		√		√	
United Arab Emirates	√		√			√
United Kingdom	√		√		√	
United States of America	√		√		√	

*HDI, human development index. The HDI was ranked “very high,” “high,” “medium,” and “low” in each country by the UNDP. In this study, we set the original “very high” and “high” rank as the “High HDI group” and “medium” and “low” as the “Low HDI group” in the subgroup analysis.*

*This study categorized the countries with a higher dentist density than the median of sum into the high dentist density group. The mean dentist density equals 5.*

*This study categorized the countries with a population over 65 years old above 14% to be aged countries*.

### Heterogeneous Effects of COVID-19 on Different Dental Care Utilization Needs

**Figure 2:**
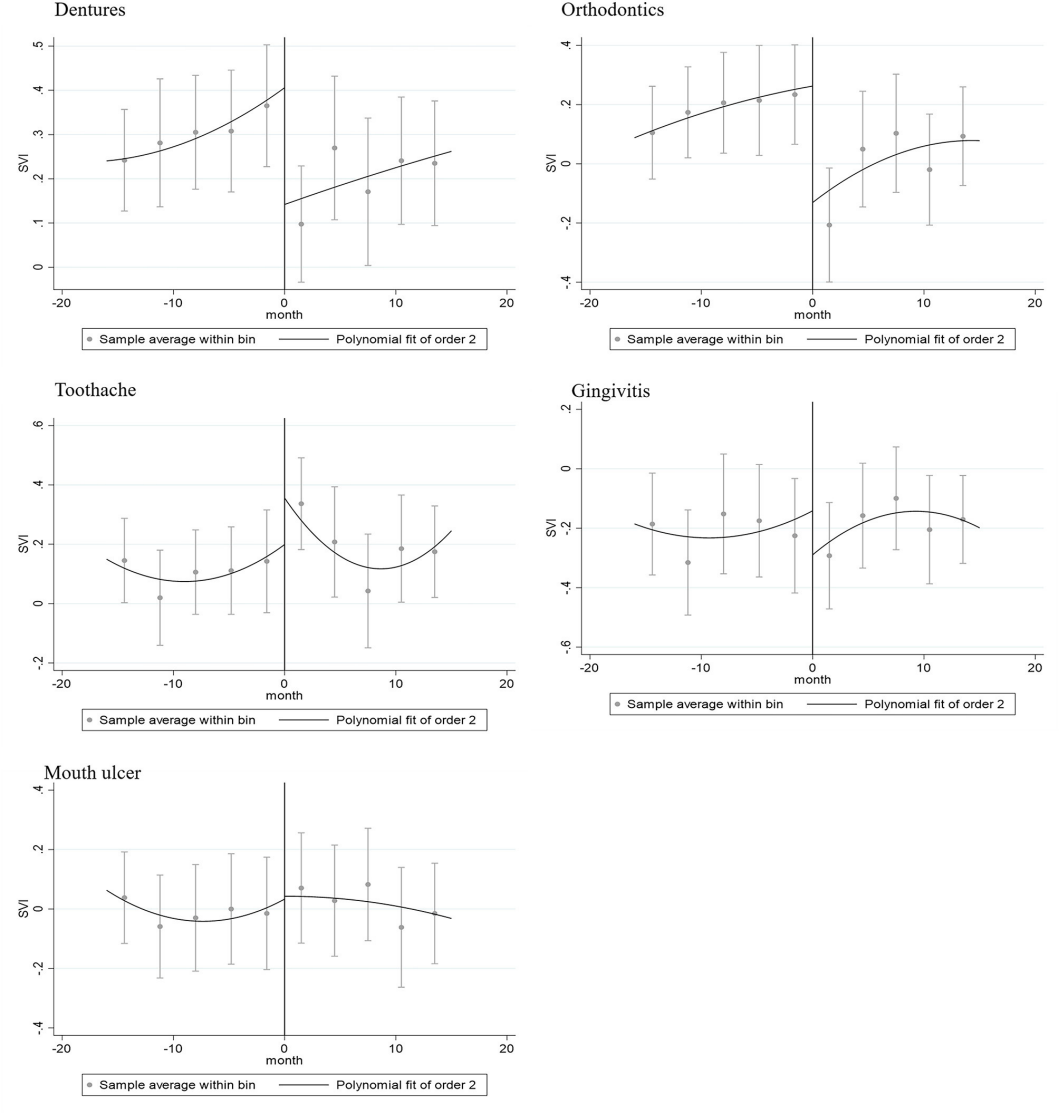
Regression discontinuity plot of topics of dentures, orthodontics, toothache, gingivitis, and mouth ulcer in March 2020.

First, we separately analyzed the effect of COVID-19 on each specific dental care in all sampled countries. Table 3 shows regression continuity estimates using the residual RSV (log) as the dependent variable, adjusted by the fixed effect of month and time trend. The RSV of “dentures” (OR: 0.60, 95% CI: 0.37–0.94) and “orthodontics” (OR: 0.63, 95% CI: 0.40–0.97) were significantly reduced during the bandwidth. Comparatively, the RSV of “toothache” (OR: 1.54, 95% CI: 0.99–2.37) was significantly increased in the COVID-19 mitigation measures at a significance level of 0.1. RSV of “gingivitis” (OR: 0.86, 95% CI: 0.52–1.42) and “mouth ulcer” (OR: 1.06, 95% CI: 0.59–1.89) had no clear discontinuities. Such an RD plot is reported in Figure 2. Figure 2 plots the discontinuities of the RSV, conditional on the timing of the COVID-19 mitigation measures. The curves were the averages of the logarithmic value of RSV across bandwidth bins of *X* to the left and right of the cutoff. The vertical solid lines are the predicted outcomes and associated confidence intervals based on a polynomial regression. Following Gelman and Imbens (40), we considered a quadratic polynomial in *X* in the baseline specification. The bandwidth was approximately 4 months. The sensitivity analysis of bandwidth is shown in Supplementary Figure S1. The discontinuity in the predicted RSV at the cutoff equals the changeable effects of RSV before the cutoff.

**Table 3:** Regression continuity estimates of COVID-19 on five dental care searching topics.

	**Advanced dental care**		**Basic dental care**		
	**Denture**	**Orthodontics**	**Toothache**	**Gingivitis**	**Mouth ulcer**
**In all samples**
OR	0.60**	0.63**	1.54*	0.86	1.06
95% CI	0.37–0.94	0.40–0.97	0.99–2.37	0.52–1.42	0.59–1.89
*P*-Value	<0.05	<0.05	<0.1	0.56	0.847
Bandwidth	3.93	4.52	3.83	6.57	5.73

The RSV of “dentures” (OR: 0.60, 95% CI: 0.37–0.94) and “orthodontics” (OR: 0.63, 95% CI: 0.40–0.97) were significantly reduced during the bandwidth. Comparatively, “toothache” (OR: 1.54, 95% CI: 0.99–2.37) were significantly increased in the COVID-19 mitigation measures at a significance level of 0.1. RSV of “gingivitis” (OR: 0.86, 95% CI: 0.52–1.42) and “mouth ulcer” (OR: 1.06, 95% CI: 0.59–1.89) had no clear discontinuities. ***P <0.01, **P <0.05, *P <0.1.

### Impact of COVID-19 Mitigation Measures on Categorized Dental Care Needs

Then, we analyzed the impact of COVID-19 on all types of dental health care and basic and advanced dental care needs in 2020 and in placebo time 2019 in all sampled countries separately. First, *T*-tests showed that there was no significant RSV difference before 12 and 12 months after the onset of COVID-19 (Table 4), which indicated that the 4-month fluctuation of RSV was associated with COVID-19 mitigation measures. In addition, after adjusting for country and disease fix effects (Table 5), advanced dental care significantly declined (aOR: 0.63, 95% CI: 0.47–0.85) due to COVID-19 mitigation measures, while there was no significant change in placebo time in 2019. The corresponding RD plots are reported in Supplementary Figure S2. The effect of COVID-19 on advanced dental care and basic dental care in different countries is shown in Supplementary Figure S3.

**Table 4:** Differences of RSV between pre-pandemic period with the pandemic period (12 months before and 12 months after onset) using *T*-Test.

	**Pre-onset**	**Post-onset**	**Mean difference**	***P*-Value**
	**Mean** **(SD)**	**Mean** **(SD)**		
Five-diseases	17.83 (17.25)	18.45 (19.57)	−0.61 (−2.22 to 0.98)	0.45
Basic diseases	15.38 (14.24)	16.67 (16.92)	−1.29 (−3.04 to 0.46)	0.14
Advanced diseases	21.49 (20.45)	21.10 (22.74)	0.39 (−2.27 to 3.37)	0.79
Toothache	22.95 (17.26)	26.71 (21.75)	−3.75 (−7.58 to 0.06)	<0.1
Gingivitis	10.91 (10.36)	11.11 (10.30)	−0.20 (−2.21 to 1.81)	0.84
Mouth ulcer	12.29 (10.85)	12.20 (11.39)	0.08 (−2.08 to 2.25)	0.93
Denture	17.29 (13.79)	17.50 (15.67)	−0.20 (−3.08 to 2.67)	0.70
Orthodontic	25.70 (24.76)	24.70 (27.67)	1.00 (−4.11 to 6.11)	0.93

**Table 5:** Estimates coefficients of regression discontinuity of dental care SVIs at baseline and after adjusting fix effect of country and disease.

	**Baseline estimates**			**Adjusted by country and disease fix effect**		
	**All type**	**Basic**	**Advanced**	**All type**	**Basic**	**Advanced**
		**dental care**	**dental care**		**dental care**	**dental care**
Observations	13,512	7,984	5,528	13,512	7,984	5,528
**COVID-19 and index (crude)**						
SVI (log)	0.91	1.09	0.69*	0.91	1.08	0.66***
95% CI	0.67–1.24	0.75–1.59	0.44–1.08	0.73–1.15	0.81–1.43	0.49–0.90
*P*-Value	0.57	0.39	<0.10	0.45	0.58	<0.001
Bandwidth (month)	5.39	9.16	6.34	5.54	9.21	5.97
**COVID-19 and index (adjusted by fix effect of month and time trend)**						
SVI (log)	0.87	1.06	0.66*	0.87	1.03	0.63***
95% CI	0.65–1.18	0.72–1.56	0.42–1.04	0.69–1.09	0.76–1.39	0.47–0.85
*P*-Value	0.39	0.74	<0.10	0.24	0.83	<0.001
Bandwidth (month)	6.03	7.68	6.74	5.26	6.90	5.91
**Placebo tests (in March 2019)**						
SVI (log)	1.02	0.99	1.11	1.04	1.02	1.06
95%CI	0.77–1.35	0.70–1.41	0.70–1.76	0.85–1.26	0.77–1.34	0.80–1.41
*P*-Value	0.87	0.97	0.65	0.72	0.87	0.66
Bandwidth (month)	10.92	10.04	13.10	9.27	8.70	10.75

Quantitative measures of COVID-19 impact on basic dental care and advanced dental care were reported in this table. We firstly analyzed the association between COVID-19 and SVIs without controlling any other effect, the coefficients in baseline estimates were not significant. After adjusting the fix effect of country and dental care type, lower SVIs of advanced dental care was associated with COVID-19 mitigation measures (OR: 0.66, 95%CI: 0.49–0.90). Then we controlled the fix effect of month and time trend, lower SVIs of advanced dental care was associated with COVID-19 mitigation measures (OR: 0.63, 95%CI: 0.47–0.85) after controlling the fix effect of country and disease. Column 1–3 showed the RD estimates without taking any treatment on logarithmic value of SVIs, column 4–6 showed the RD estimates after adjusting fix effect of country and disease. Comparatively, there was no statistically significant difference of RD estimates in the placebo test. ***P <0.01, **P <0.05, *P <0.1.

### Subgroup Analysis in Categorized Countries With Different HDI Ranks, Dentist Densities, and Age Structures

In the countries with a high HDI rank (Table 6), the RSV of dentures (OR: 0.61, 95% CI: 0.37–0.97) were significantly reduced, while the RSV of toothaches (OR: 1.61, 95% CI: 1.07–2.39) were increased. The RSV of dentures (OR: 0.55, 95% CI: 0.31–0.97) and orthodontics (OR: 0.61, 95% CI: 0.38–0.96) were significantly reduced, and toothache was significantly increased (OR: 1.85, 95% CI: 1.14–3.01) in countries with high dentist density. The RSV of orthodontics (OR: 0.61, 95% CI: 0.34–1.08) and gingivitis (OR: 0.56, 95% CI: 0.31–0.98) were significantly reduced in countries with a low percentage of the aged population. However, all the RSVs of toothache increased in all types of countries, with different significance. The corresponding RD plot was reported in appendix Supplementary Figure S4.

**Table 6:** Regression continuity estimates of COVID-19 on five dental care keywords in countries with different backgrounds.

	**Advanced dental care**		**Basic dental care**		
	**Denture**	**Orthodontics**	**Toothache**	**Gingivitis**	**Mouth ulcer**
**Categorized by HDI rank**					
**Low HDI**					
OR	0.74	0.59	1.36	0.67	1.30
95% CI	0.41–1.29	0.29–1.16	0.55–3.37	0.39–1.15	0.37–4.46
*P*-Value	0.28	0.12	0.67	0.15	0.41
Bandwidth	6.03	4.31	2.90	6.35	7.68
**High HDI**					
OR	0.63*	0.61**	1.61**	1.02	1.08
95% CI	0.37–1.05	0.37–0.97	1.07–2.39	0.51–2.01	0.55–2.09
*P*-Value	<0.1	<0.05	<0.05	0.96	0.82
Bandwidth	4.00	3.14	3.55	5.77	3.71
**Categorized by Dentists Density**					
**Countries with density** ** <5 (medium) per 1,000 persons**					
OR	0.65*	0.61	1.19	0.76	0.88
95% CI	0.40–1.05	0.32–1.14	0.65–2.17	0.48–1.17	0.38–2.01
*P*-Value	<0.1	0.12	0.56	0.21	0.76
Bandwidth	6.49	4.96	9.85	5.02	6.60
**Countries with density** **≥5 per 1,000 persons**					
OR	0.55**	0.61**	1.85***	1.04	1.28
95% CI	0.31–0.97	0.38–0.96	1.14–3.01	0.44–2.47	0.60–2.70
*P*-Value	<0.05	<0.05	<0.01	0.92	0.51
Bandwidth	3.63	5.32	3.82	5.85	4.82
**Categorized by age structure**					
**Countries with aged 65** **<** **14%**					
OR	0.44	0.61*	1.22	0.56**	1.17
95% CI	0.15–1.26	0.34–1.08	0.69–2.15	0.31–0.98	0.36–3.69
*P*-Value	0.12	<0.1	0.49	<0.05	0.79
Bandwidth	2.74	3.42	3.60	2.73	3.65
**Countries with age 65** **≥14%**					
OR	0.56**	0.58***	1.43	0.91	1.00
95% CI	0.31–1.00	0.37–0.87	0.68–2.97	0.41–2.03	0.45–2.19
*P*-Value	<0.05	<0.01	0.34	0.82	0.99
Bandwidth	3.77	3.07	5.83	6.43	5.35

***P <0.01, **P <0.05, *P <0.1.

## Discussion

This primary finding of this study showed that the RSV of toothache increased by 1.54 times in approximate 4 months after the COVID-19 mitigation implementation in March 2020, while the need for orthodontics and dentures decreased by nearly 40% among 17 countries in the world, using Google Trends searching topics with a quasi-experimental analysis. Such trends are more evident in countries with a high HDI and high dentist density than in countries with a lower HDI and low dentist density.

This finding observed that the sudden effect time of COVID-19 mitigation measures on global dental health care needs lasted about 4 months, which means such online dental care searching interest fluctuation lasted till July, 2020. The results were consistent with but more generalized than those of previous studies in estimating the effect of COVID-19 on dental care utilization. A study from the United States showed that in the first few days of June, 71% of dental clinics were open, but the number of patients was less than usual (41). By June 20, the number of weekly visits rebounded sharply (42). Another survey indicated that 71.1% of dentists would reopen dental clinics on May 20, 2020 (43). The present findings indicated that the estimated effect duration of the COVID-19 mitigation measure on dental care needs was longer than expected. Compared to cardiovascular diseases and diabetes that recovered in early April 20 (44), the recovery time of dental care needs seems to be longer than that of other severe chronic diseases. This may be related to the fact that a lower priority of dental care was in most people's minds, and it is believed that dental disease is not harmful to general health compared to severe chronic diseases. In the present study, the estimation of the time discontinuity of dental care needs is essential for all stakeholders when confronting a similar situation because of the continuous impact of COVID-19, such as new wave outbreaks caused by the Omicron Variant at the end of 2021 (45).

The findings of this study identified the heterogeneous effect of COVID-19 mitigation measures on different dental care categories. The search term “toothache” was significantly sharply increased after mitigation of COVID-19, which was consistent with previous studies (17, 19). The sharp increase in the need for emergency dental care may be related to stress, changes in diet patterns and oral hygiene behaviors during the pandemic, and delayed care (46). These changes predicted that the burden of untreated dental diseases would increase rapidly in a short time (47, 48). Dental professionals should be prepared to face the increasing demand for emergency dental care and adverse clinical outcomes in a short time after the outbreak of the COVID-19 epidemic (49). Comparatively, this finding observed a similar decline in the need for orthodontics (50). This phenomenon could be explained by the different motivations for searching activities online in the context of the pandemic. The decline in orthodontics and dentures may be related to the lower priority of non-urgent oral health during the pandemic. Dental professionals who specialized in such fields should prepare for facing short term financial stress of clinics operation.

Interestingly, COVID-19 mitigation had a greater effect on increasing toothache in countries with a high human development index and high dentist density. This phenomenon indicated that the prevalence of dental disease onset, especially emergency dental diseases, did not decline even in countries with better socioeconomic backgrounds. This may be related to the traditional dental care always focused on treating symptoms of dental diseases rather than emphasizing prevention (51). Shifting more toward prevention-centric approaches to care and away from surgical interventions should be considered in the future (52). This finding also reflect the socioeconomic disparities of dental care that are profoundly persistent in the world (53). People from countries with a high human development index and high dentist density may have more conveniently routine dental care accessibility and better oral health perception. Their urgent dental needs could be instantly satisfied due to sufficient dental care supplies before mitigation (54). Therefore, they felt more affected and had more online dental health information seeking behaviors during the pandemic. This may be related to the existing inequalities and heterogeneity in dental care between countries rather than the lower priority of oral health care during the pandemic. The COVID-19 pandemic seems to have revealed such inequalities (53). Eliminating socioeconomic disparities in dental care should be considered when developing public health strategies. In a short period, dental professionals should implement different health management strategies for primary dental care and advanced dental care during the lockdown period to satisfy different dental needs. Increasing preventive dental intervention to reduce acute dental disease should be persistently encouraged in the long run. Essential oral health care should be given priorities according to specific development status in countries when considering oral health care in the primary care system (30).

There are several limitations to this study. First, countries that do not use Google or lack sufficient data are excluded. Therefore, these results may reflect a potential trend of countries using Google, but they are not representative of all countries. Second, the RSV of dental care can provide us with a broad perspective to analyze real-time global health care demand. However, due to repeatedly searching of the same user, RSV may be higher than the actual dental needs. Third, we used five specific dental diseases to represent the different dental care, which only represents a part of dental diseases. Other dental diseases may be overlooked in the analysis. Future research may consider using an extensive database to evaluate more accurate results. Fourth, we assessed the short-term impact on dental health. Considering that the pandemic is still ongoing, researchers should use longitudinal data to evaluate the long-term effect on dental care. Future studies need to summarize our findings.

Despite these limitations, this research shows several advantages. First, we used a quasi-experimental method to estimate the causal effect of COVID-19 on global dental care. To the best of our knowledge, this is the first article to analyze the causal impact of COVID-19 mitigation measures on global dental care needs by using Internet search data. Second, we gave quantitative estimates of the sudden impact of COVID-19 on dental care. The estimates, including the duration and a specific disease, could provide a reference for the recovery time after the pandemic. By using this data-driven estimation time, dentists could prepare for shutdown hours and reduce economic pressure (55). Third, health care decision-makers could redistribute different dental resources to reduce harmful consequences caused by the unexpected pandemic on the dental care market.

## Conclusion

The influence of COVID-19 mitigation measures was different in dental care categories and different countries. The mitigation measure of COVID-19 exposed the existing disparities in dental care utilization and insufficient preventive dental care. Eliminating socioeconomic disparities in dental care should be considered when developing public health strategies.

## Data Availability Statement

The datasets presented in this study can be found in online repositories. The names of the repository/repositories and accession number(s) can be found below: Dental care SVIs are available at https://trends.google.com/trends/?geo=US. HDI rank is available at http://hdr.undp.org/en/content/human-development-index-hdi. Dentist density is available at https://www.who.int/data/gho/data/indicators/indicator-details/GHO/dentists-(per-10-000-population). Age structures are available at https://data.worldbank.org/indicator/SP.POP.1564.TO.ZS.

## Author Contributions

XQ contributed to the conception, design, data acquisition, analysis, interpretation, drafted, and critically revised the manuscript. CXY contributed to the analysis and critically revised the manuscript. QYH and ZRL contributed to the analysis and interpretation and critically revised the manuscript. SH contributed to the conception, data interpretation, drafted, and critically revised the manuscript. WZ contributed to the conception, acquisition and analysis, and critically revised the manuscript. DL contributed to the design, acquisition, drafted the manuscript, and critically revised the manuscript. All authors gave final approval and agree to be accountable for all aspects of the work ensuring integrity and accuracy.

## Funding

This study was supported by the National Natural Science Foundation of China, No. 71904136 and the Sichuan Science and Technology Agency, No. 2020YFS0582. The funding organization had no role in the study design, implementation, analysis, or interpretation of the data.

## Conflict of Interest

The authors declare that the research was conducted in the absence of any commercial or financial relationships that could be construed as a potential conflict ofinterest.

## Publisher's Note

All claims expressed in this article are solely those of the authors and do not necessarily represent those of their affiliated organizations, or those of the publisher, the editors and the reviewers. Any product that may be evaluated in this article, or claim that may be made by its manufacturer, is not guaranteed or endorsed by the publisher.

## Abbreviations

COVID-19: coronavirus disease 2019
RD: regression discontinuity
RSV: relative search volume
HDI: human development index
OR: Odds ratios
CI: confidence intervals.
