# Supplementary material for: Effect of the COVID-19 Mitigation Measure on Dental Care Needs in 17 Countries: A Regression Discontinuity Analysis
Source: Front Public Health. 2022 May 31;10:890469. doi: 10.3389/fpubh.2022.890469 (PMC9194817; doi:10.3389/fpubh.2022.890469)

**Supplementary**

# Effect of the COVID-19 Mitigation Measure on Dental Care Needs in Seventeen Countries: A Regression Discontinuity Analysis

Xing Qu^1^, Chenxi Yu^2^, Qingyue He^3^, Ziran Li^4^, Shannon H. Houser^5^, Wei Zhang^1,6*^, Ding Li^7*^

^1^ Institute of Hospital Management, West China Hospital, Sichuan University, Chengdu, China; ^2^ College of Economics and Management, Sichuan Normal University, Chengdu, China; ^3^ Southwest Medical University, Chengdu, China; Center of Health Care Management, Chengdu First People’s Hospital, Chengdu Integrated TCM & Western Medicine, Chengdu, China; ^4^ School of Public Finance and Taxation, Southwestern University of Finance and Economics, Chengdu, China; ^5^ Department of Health Services Administration, University of Alabama at Birmingham, Alabama, USA; ^6^ West China Biomedical Big Data Center, Med-X Center for Informatics, Sichuan University, Chengdu, China; ^7^ Institute of Development Studies, Southwestern University of Finance and Economics, Chengdu, China.

**Corresponding author: Wei Zhang,** [weizhanghx@163.com](mailto:weizhanghx@163.com), +86-18980601010;

**Co-corresponding author:** Ding Li, [liding@swufe.edu.cn](mailto:liding@swufe.edu.cn), +86-13980676565

**Supplementary S1: Figure of sensitivity to Bandwidth in the Continuity-Based Approach**

Note：This figure indicates the local average treatment estimates and robust 95% confidence interval using different methods to evaluate bandwidth.

We illustrate the sensitive approach for four bandwidth choices close to MSE-optimal and CER-optimal，the line from left to right represents (i) the CER-optimal choice: h-CER; (ii) the MSE-optimal choice h-MSE, (iii) the double CER-optimal bandwidth: 2·h-CER; (iv) the double MSE-optimal bandwidth: 2·h-MSE

**Supplementary S2:** RD plots of regression discontinuity of dental care SVIs at baseline and after adjusting fix effect of country and disease


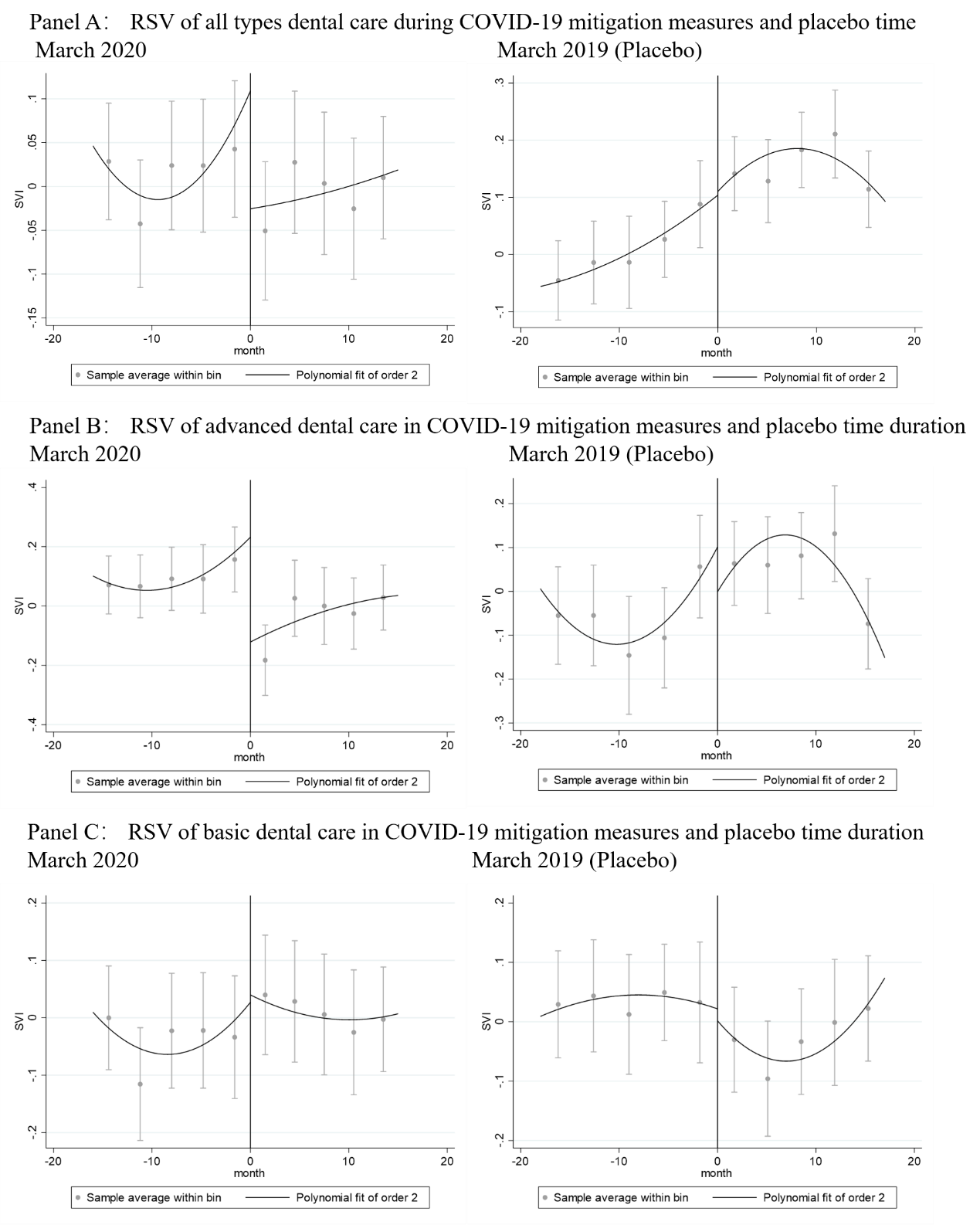


**Supplementary S3:** Effect of COVID-19 on the advanced dental care and basic dental care in different countries


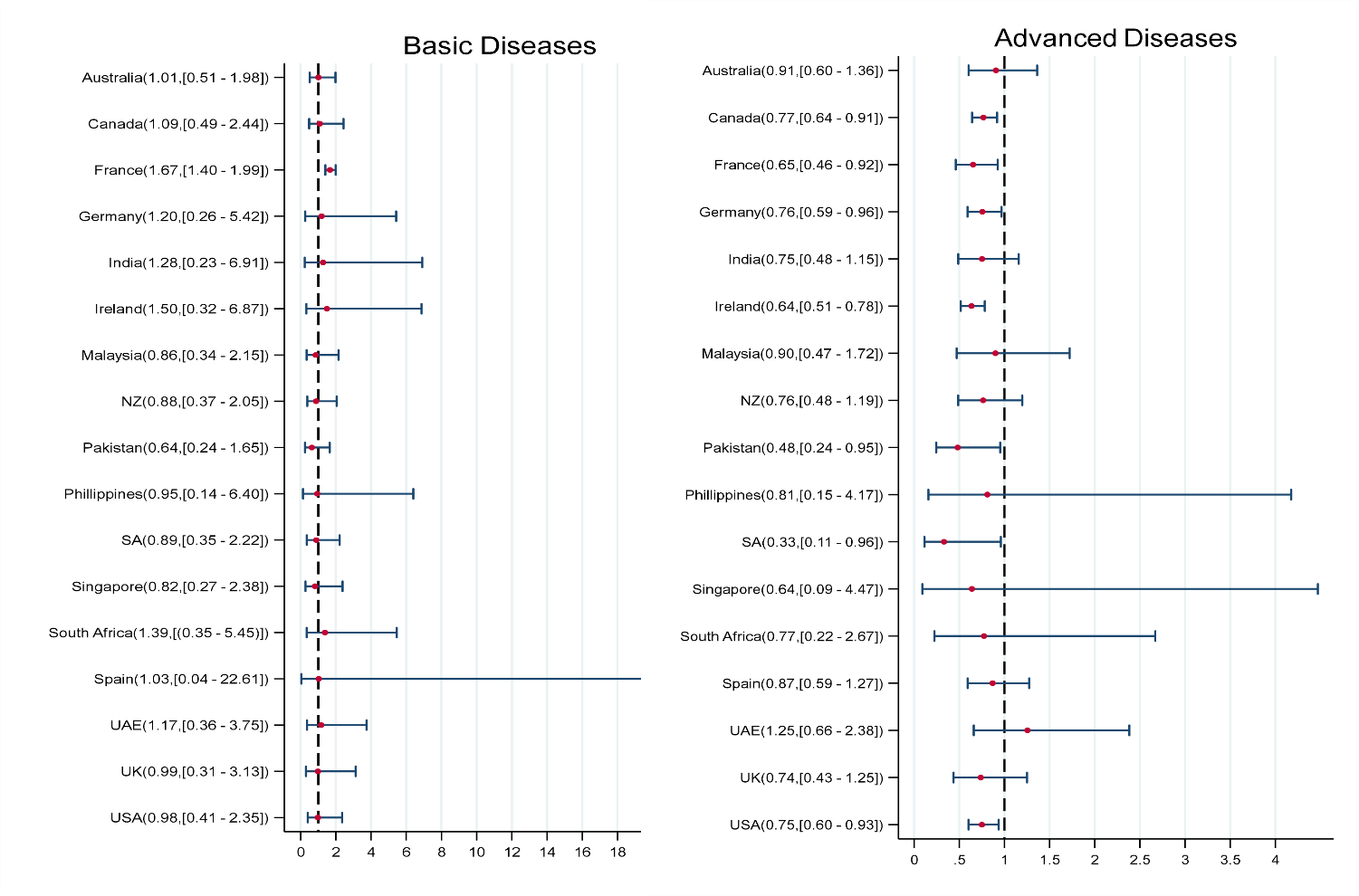


**Supplementary S4:**

Regression continuity estimates plot of COVID-19 on five dental care keywords in countries with different backgrounds


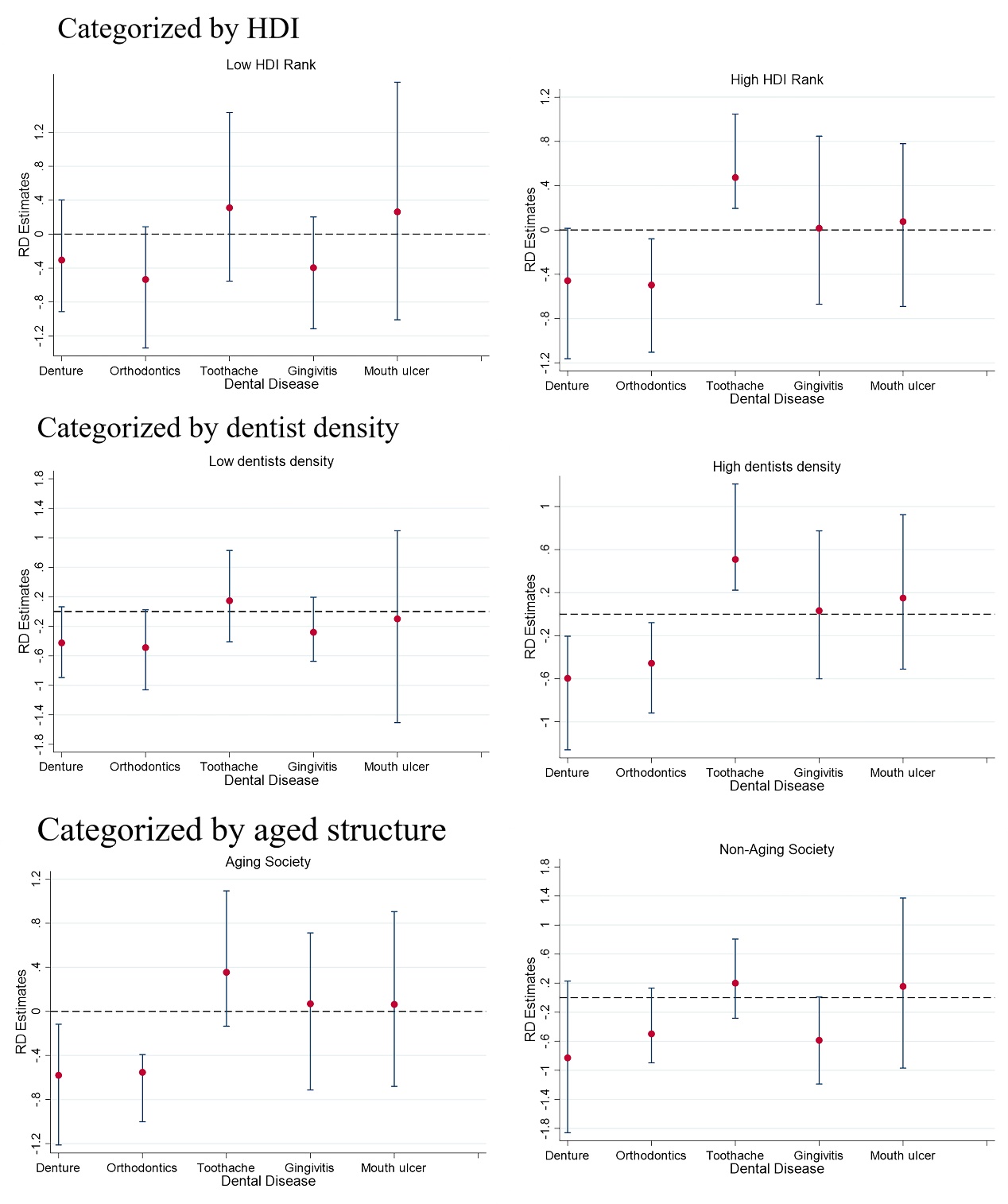

Supplement: Supplementary file 1 [file Data_Sheet_1.DOCX]
